# Prenatal diagnosis of Fraser syndrome caused by novel variants of *FREM2*

**DOI:** 10.1038/s41439-020-00119-5

**Published:** 2020-10-02

**Authors:** Shoko Ikeda, Chika Akamatsu, Akifumi Ijuin, Ami Nagashima, Megumi Sasaki, Akihiko Mochizuki, Hiromi Nagase, Yumi Enomoto, Yukiko Kuroda, Kenji Kurosawa, Hiroshi Ishikawa

**Affiliations:** 1 Department of Obstetrics and Gynecology, Kanagawa Children’s Medical Center, Yokohama, Japan; 2 Clinical Research Institute, Kanagawa Children’s Medical Center, Yokohama, Japan; 3 Division of Medical Genetics, Kanagawa Children’s Medical Center, Yokohama, Japan

**Keywords:** Disease genetics, Genetics

## Abstract

Fraser syndrome (FS) involves multiple malformations and has a 25% recurrence risk among siblings. However, these malformations are difficult to detect prenatally, hampering prenatal diagnosis. Here, we describe a fetus with FS diagnosed using ultrasonography. Ultrasonography revealed congenital high airway obstruction syndrome and renal agenesis. Syndactyly of both hands and cryptophthalmos were noted postnatally, and the diagnosis was confirmed by genetic analysis, which showed novel compound heterozygous variants of *FREM2*.

Fraser syndrome (FS; cryptophthalmos-syndactyly syndrome; OMIM #219000) is a rare autosomal recessive multiple malformation syndrome characterized by cryptophthalmos, syndactyly, and respiratory and urogenital tract anomalies^1–4^. Over 250 cases have been reported to date^2^. The prevalence of FS is 0.43 per 100,000 and 11.06 per 100,000 among newborns and stillborns, respectively^5^. FS is a genetically heterogeneous condition, and pathogenic variants of *FRAS1* and *FREM2*, which encode a family of large extracellular matrix (ECM) proteins, are implicated in the etiology of FS. These proteins are essential for the adhesion of epidermal basement membranes to dermal connective tissues during embryogenesis^6,7^. Because anomalies such as fused eyelids and those in the digits, larynx, and vagina result from failed programmed cell death, defective apoptosis seems to be the key mechanism underlying FS. Recently, pathogenic variants of *GRIP1*, which encodes glutamate receptor-interacting protein 1, have also been identified to cause FS in humans^8^.

The current clinical diagnostic criteria proposed by van Haelst et al. include six major manifestations (syndactyly, cryptophthalmos spectrum, ambiguous genitalia, urinary tract abnormalities, laryngeal and tracheal anomalies, and positive family history) and five minor symptoms (anorectal defects, dysplastic ears, skull ossification defects, umbilical defects, and nasal anomalies)^2^. The authors suggested that a diagnosis of FS can be made if either three major, two major and two minor, or one major and three minor symptoms are present in a patient.

As FS is an autosomal recessive disease and has a recurrence risk of 25% among siblings, both prenatal and postnatal diagnoses are important in affected families. However, major manifestations, such as cryptophthalmos and syndactyly, are difficult to detect via prenatal ultrasonography, especially in the presence of oligohydramnios. Therefore, few prenatal cases have been reported.

Here, we report a case of FS prenatally suspected at 19 weeks of gestation and diagnosed via genetic analysis, which uncovered novel compound heterozygous *FREM2* variants. This study was approved by the Institutional Review Board of Kanagawa Children’s Medical Center, and clinical information was collected after obtaining written informed consent from the parents.

A 38-year-old female, gravida 2, para 0, was referred to our hospital because of oligohydramnios and fetal ascites at 19 weeks of gestation. The parental history of the fetus was unremarkable, and consanguinity was absent. Ultrasonography revealed severe oligohydramnios, bilateral renal agenesis, no urinary bladder, and numerous ascites (Fig. 1a, b). Head and long-bone biometry results were normal; however, the abdomen was distended owing to ascites and hepatomegaly. The lungs were enlarged and hyperechogenic, with an inverted diaphragm (Fig. 1c). The trachea was distended from the caudal of the carina to the bronchi (Fig. 1d). The heart and intracranial structures were normal. The digits of the hands and feet were barely visible. The enlarged lungs, distended trachea, and ascites indicated congenital high obstruction airway syndrome (CHAOS). The concurrence of CHAOS and renal agenesis prompted us to suspect FS, which met two major criteria^2^. Given the poor prognosis, the parents terminated the pregnancy at 21 weeks. A male infant was delivered with a birth weight of 520 g and a length of 29.5 cm. His facial features included bilateral cryptophthalmos and low-set, malformed ears (Fig. 1e, f). Syndactyly of both hands (I–IV) was also noted (Fig. 1g). Autopsy revealed pleural effusion and ascites, atresia of the epiglottis with a dilated trachea, hyperinflated and heavy lungs, and agenesis of the kidneys, ureters, and bladder. Chromosomal analysis revealed a normal male karyotype.

**Fig. 1:**
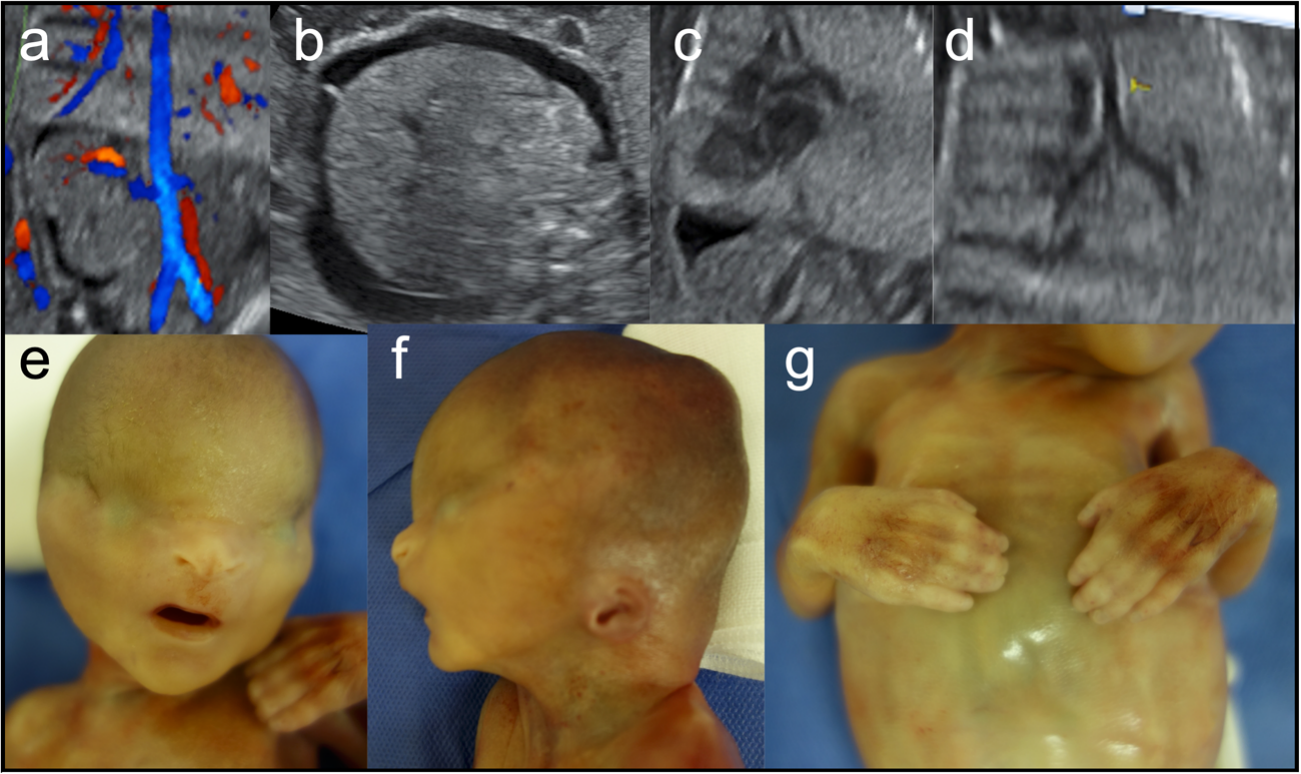
Prenatal ultrasonography images and postnatal diagnosis of a fetus with Fraser syndrome. Bilateral renal agenesis: color Doppler image of the renal artery (**a**). Severe ascites (**b**), a hyperechogenic lung with an inverted diaphragm (**c**), and a distended trachea (**d**) were observed at 19 weeks of gestation. Fetal facial features included cryptophthalmos, hypertelorism, a small, low-set nose with a bifid tip, and microtia (**e**, **f**). Bilateral cutaneous syndactyly of the hands was also observed (**g**).

Genomic DNA was extracted from the umbilical cord blood of the fetus and peripheral blood of the parents using a QIAcube Kit (QIAGEN, Hilden, Germany). Targeted sequencing was performed using a TruSight One Sequencing Panel (Illumina, San Diego, CA, USA) on the MiSeq platform (Illumina) with 151-bp paired-end reads, as previously described^9^. Candidate variants were subsequently confirmed by Sanger sequencing.

We identified the compound heterozygous variants: NM_207361.5:c.3628_3628dupA:p.(Thr1210Asnfs*8) and c.6203 T > A:p.(Leu2068*) of *FREM2*. Sanger sequencing confirmed that the c.3628_3628dupA variant was inherited from the father, whereas the c.6203 T > A variant was inherited from the mother (Fig. 2). These variants are novel and absent in gnomAD (https://gnomad.broadinstitute.org/), ClinVar (https://www.ncbi.nlm.nih.gov/clinvar/), jMorp (https://jmorp.megabank.tohoku.ac.jp/202001/variants), the Human Genetic Variation Database (http://www.hgvd.genome.med.kyoto-u.ac.jp/), and the Human Genome Mutation Database 2019.4 (https://portal.biobase-international.com/). Both *FREM2* variants are predicted to contain premature termination codons, consistent with biallelic loss-of-function.

**Fig. 2:**
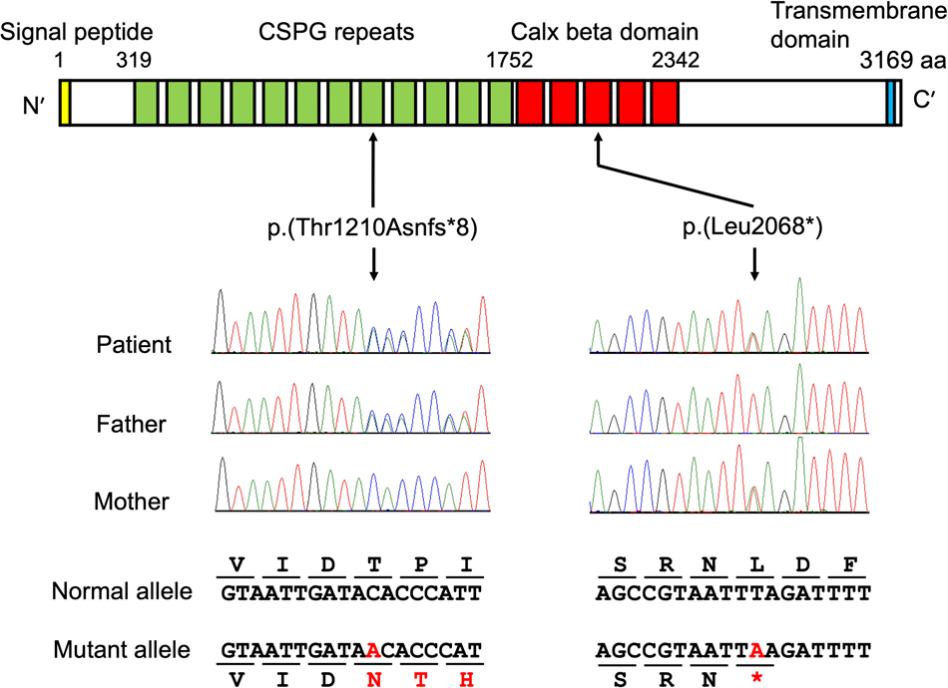
Schematic of FREM2 protein structure and compound heterozygous *FREM2* variants. FREM2 contains chondroitin sulfate proteoglycan (CSPG) repeats and the calcium-binding loop of Na^2+^–Ca^2+^ exchange (Calx) beta domains (https://www.uniprot.org/). Electropherograms revealed biallelic variants, c.3628_3628dupA:p.(Thr1210Asnfs*8) and c.6203 T > A:p.(Leu2068*), derived from the father and mother of the patient, respectively.

*FRAS1* mutations are the most common cause of FS, accounting for 65% of cases, while *FREM2* mutations account for 20% of cases, and *GRIP1* is associated with a limited number of cases^8,10^. Thus, definitive diagnosis is difficult in families without prior affected siblings. To date, only two prenatally diagnosed FS cases without any family history have been reported^11,12^. To our knowledge, this is the first report of a Japanese FS patient carrying *FREM2* pathogenic variants.

FRAS1 and FREM2 are multidomain ECM transmembrane proteins, and GRIP1 is required for the presentation of the FRAS/FREM complex at the basal membrane of epithelial cells. ECM proteins are important for cell adhesion and migration, basement membrane integrity, and epithelial-mesenchymal interactions during development^6,7,13^. Metanephric kidney development depends on interactions between the ureteric bud epithelium and adjacent mesenchyme, during which ECM proteins are involved^14^. Cryptophthalmos and syndactyly arise from the loss of epidermal adhesion, causing interrupted epidermal/mesenchymal interactions between the eyelid epithelia or limb apical ectodermal ridge and the underlying mesenchyme^15^.

Ultrasonography is an important tool for the prenatal diagnosis of malformations. Teisser et al. analyzed 38 pre- and postnatal cases (of which 36 had a family history) and observed bilateral or unilateral renal agenesis and airway obstruction in 61 and 42% of the fetuses, respectively, by ultrasonography^3^. The same authors observed eye anomalies, including microphthalmia or hypertelorism, and syndactyly in only 15 and 8% of the cases, respectively. Laryngeal atresia is a common cause of CHAOS and involves large echogenic lungs, a flattened or inverted diaphragm, dilated airways below the level of the obstruction, and fetal ascites or hydrops. Fetuses with CHAOS often exhibit polyhydramnios, but in FS, CHAOS with oligohydramnios can occur because of severe renal insufficiency^16^. Our patient was referred because of fetal ascites. To determine the cause, we investigated the fetus in detail using ultrasonography and discovered that laryngeal atresia was the cause of ascites. This satisfied two major criteria of FS, strongly suggesting this diagnosis. As in the present case, the concurrence of renal agenesis and laryngeal atresia is recognized as a valuable sonographic marker of FS, especially in patients without affected siblings^3,10,17,18^. In routine ultrasound examinations, the coexistence of fetal hydrops (including isolated ascites) and oligohydramnios seems to indicate a differential diagnosis of FS. Additionally, syndactyly, abnormal genitalia or dysplastic ears may facilitate the diagnosis of FS via ultrasonography^3,11^. Kornacki et al. reported a case of FS that showed CHAOS, a suspicion of syndactyly, and orbit asymmetry in prenatal ultrasonography; the diagnosis was confirmed by postnatal genetic analysis^19^. However, we did not detect these features in a subsequent fetal scan owing to severe oligohydramnios.

In conclusion, we identified novel compound heterozygous variants of *FREM2* in a prenatal FS case diagnosed via ultrasonography, which is difficult but possible for fetuses exhibiting a combination of cardinal features, even if there is no affected sibling. Additionally, despite the marked interfamilial clinical heterogeneity of FS, strong phenotypic similarities exist within families^2,20^. Postmortem examinations of fetal and neonatal cases, followed by genetic analyses, may confirm diagnoses and consequently improve genetic counseling, prenatal molecular diagnosis, and sonographic screening during subsequent pregnancies.

## Data Availability

The relevant data from this Data Report are hosted at the Human Genome Variation Database at 10.6084/m9.figshare.hgv.2882 10.6084/m9.figshare.hgv.2885
